# Recruitment and Retention Strategies of Cancer Patients and Their Caregivers in Family-based Intervention Studies

**DOI:** 10.1093/geroni/igaa057.3349

**Published:** 2020-12-16

**Authors:** Ting Guan, Yousef Qan’ir, Ahrang Jung, Shenmeng Xu, Eno Idiagbonya, Lixin Song

**Affiliations:** 1 University of North Carolina at Chapel Hill, Chapel Hill, North Carolina, United States; 2 University of North Carolina at Chapel Hill, Chapel hill, United States; 3 University of North Carolina at Chapel Hill, Chapel hill, North Carolina, United States

## Abstract

Family-based psychosocial behavioral interventions (PBIs) that target both the cancer patients and their caregivers may more effectively help them with self-care and improve quality of life; however, family-based PBIs often face unique challenges during study implementation. This systematic review aimed to a) examine the recruitment and retention rates of cancer patients and their caregivers in clinical trials testing family-based PBIs; and b) explore the recruitment and retention strategies. We systematically searched five electronic databases to identify randomized controlled trials that tested family-based psychosocial or behavioral interventions among adult patients with cancer and their adult family caregivers. Our searches yielded 48 studies. The average recruitment rates of patients and caregivers were 56.8% (SD=31.8%; range=8-100%) and 54.5% (SD=32.4%; range=8-100%), respectively. The majority of the studies have focused on white and female patients and caregivers. The average retention rate at end of follow-up times was 69.1%. Only 13 studies reported retention strategies, including providing money/gift cards upon returning of each follow-up survey or study completion, and excluding advanced cancer patients. Reasons for attrition, i.e., dropping out of studies, were classified as: health-related (e.g., death, illness, psychological distress), intervention-related (e.g., intervention does not meet expectation, frustration with group allocation, intervention burden) and other reasons (e.g. lack of time, unable to establish contact). Recruitment and retention of patients and caregivers in family-based PBI are integral to the success of interventions. Researchers need to incorporate effective strategies for optimizing recruitment and retention at the planning stage of their studies.

